# Environmental hazard of polypropylene microplastics from disposable medical masks: acute toxicity towards *Daphnia magna* and current knowledge on other polypropylene microplastics

**DOI:** 10.1186/s43591-021-00020-0

**Published:** 2022-01-04

**Authors:** Anita Jemec Kokalj, Andraž Dolar, Damjana Drobne, Marjan Marinšek, Matej Dolenec, Luka Škrlep, Gregor Strmljan, Branka Mušič, Andrijana Sever Škapin

**Affiliations:** 1 Department of Biology, Biotechnical Faculty, University of Ljubljana, Večna pot 111, SI-1000 Ljubljana, Slovenia; 2 Faculty of Chemistry and Chemical Technology, Chair of Materials and Polymer Science, University of Ljubljana, Večna pot 113, SI-1000 Ljubljana, Slovenia; 3 Department of Geology, Faculty of Natural Sciences and Engineering, University of Ljubljana, Aškerčeva 12, SI-1000 Ljubljana, Slovenia; 4 Slovenian National Building and Civil Engineering Institute, Dimičeva ulica 12, SI-1000 Ljubljana, Slovenia

**Keywords:** COVID-19 pandemic, Plastics-associated chemicals, *Daphnia magna*, Fibres, Fragments, Nanofibres, Polypropylene microplastics, Single-use plastic

## Abstract

The COVID-19 pandemic has increased the use of disposable plastics, including medical masks, which have become a necessity in our daily lives. As these are often improperly disposed of, they represent an important potential source of microplastics in the environment. We prepared microplastics from polypropylene medical masks and characterised their size, shape, organic chemical leaching, and acute toxicity to the planktonic crustacean *Daphnia magna*. The three layers of the masks were separately milled and characterised. Each of the inner frontal, middle filtering, and outer layers yielded different types of microplastics: fibres were obtained from the inner and outer layer, but irregular fragments from the middle layer. The shape of the obtained microplastics differed from the initial fibrous structure of the intact medical mask layers, which indicates that the material is deformed during cryo-milling. The chemical compositions of plastics-associated chemicals also varied between the different layers. Typically, the inner layer contained more chemicals related to antimicrobial function and flavouring. The other two layers also contained antioxidants and their degradation products, plasticisers, cross-linking agents, antistatic agents, lubricants, and non-ionic surfactants. An acute study with *D. magna* showed that these microplastics do not cause immobility but do physically interact with the daphnids. Further long-term studies with these microplastics are needed using a suite of test organisms. Indeed, studies with other polypropylene microplastics have shown numerous adverse effects on other organisms at concentrations that have already been reported in the environment. Further efforts should be made to investigate the environmental hazards of polypropylene microplastics from medical masks and how to handle this new source of environmental burden.

**Please check the SI word document. The authors are not listed there. I cannot edit that file. Please add the authors. Supplementary Information:**

The online version contains supplementary material available at 10.1186/s43591-021-00020-0.

## Introduction

With the crisis of the global coronavirus pandemic (COVID-19), the consumption of single-use plastics, including personal protective equipment, has increased significantly. This has become essential to prevent the spread of infection among healthcare workers and the general public [1–3]. Among the protective equipment, disposable medical (face) masks (also called surgical masks) are most commonly used by the general public, as some governments have recommended or mandated their use indoors as well as outdoors [1].

The use of medical masks as an infection control measure was common in East and South-East Asia at the onset of the COVID-19 pandemic, and it then gained momentum in the rest of the world during 2020 and 2021 [4]. The production volume as well as the use of medical masks is already enormous, and is expected to continue to increase in the near future. For example, at the end of April 2020, China was producing about 450 million medical masks daily. Indeed, as the leading manufacturer, the annual production volume of medical masks in China increased from 5 billion in 2019 to 10 billion in 2020 [5].

There is a wide variety of medical masks on the market that are made of different plastics, such as polyurethane, polyacrylonite, polyester, polyethylene terephthalate and polypropylene. The last of these, polypropylene, remains by far the most common material and has a long history of use [6–8]. This was also confirmed by searching online medical devices catalogues (search term: surgical masks; Medical Expo, 2020), where all of the medical masks that indicated the polymer composition (about 20% of those available) were polypropylene. In addition, many companies online sell polypropylene as the raw material for making medical masks. Generally, medical masks consist of three main layers: the inner frontal layer, the middle filtering layer, and the outer layer, which is usually water repellent and coloured. The filter materials are produced by a ‘non-woven’ approach, which refers to the layers of the fibres as bonded together by physical entanglements or contact adhesion between the individual fibres [9, 10]. This approach includes different processes, such as for melt-blown and spun-bond fabrics, each of which results in different final fibre diameters. The most commonly reported approach to produce the middle filter layer of the medical mask material is melt blowing, while the spun-bond process is used to produce the inner and outer layers of the masks [9].

The major environmental concern associated with the increasing use of disposable medical masks by the general public is poor waste management [2, 6, 7]. Although it was suggested recently that a possible route for waste management would be thermo-chemical conversion of disposable medical masks into value-added products [11], improper disposal of masks in public spaces [2] and into the environment in general [6, 7], is widespread. This contributes to global plastics pollution, which has numerous negative impacts on the environment [12]. In addition, as identified recently, disposable medical masks might represent a significant new source of microplastics [2, 6, 7, 13, 14].

Similar to other plastic items, once medical masks are disposed of into the natural environment, they undergo weathering processes, including ultraviolet radiation, temperature fluctuations, increased humidity, biodegradation, physical abrasion and chemical oxidation. Weathering affects the physicochemical properties of plastics, and eventually leads to their fragmentation into microplastics and nanoplastics [15–19]. The degradation of plastics is highly dependent on the polymer type and any chemical additives [20, 21]. Polypropylene, the material most commonly used in the medical mask production, is susceptible to photodegradation [14, 20, 22], heat [23] and atmospheric oxygen [24]. Polypropylene is excellent in terms of water resistance, but inferior in terms of weathering resistance. Moisture accelerates the oxidative degradation of polypropylene, and consequently its melting point and thermal decomposition temperature are lowered, and its mechanical properties deteriorate [25]. In the external environment, conditions can be even more severe, such as lower (acidic) pH due to acid rain, higher (alkaline) pH due to fertilization with nitrogen compounds and accelerated decomposition of plastics, and the presence of electrolytes, due to road salting or along coastal areas. A number of studies have shown that polypropylene is susceptible to outdoor weathering [19, 26], although it is not readily biodegradable [27]. To some extent, shedding and peeling of microfibres from medical masks is also likely to occur, as has been shown for polyester textiles that release fibres into water and air during household washing and drying, and during their regular use [28]. However, the release of fibres is highly dependent on the textile structure, as there will be less release for textiles with a compact woven structure [29]. There are already some estimations on the amount of microplastics released from medical masks [13, 14]. Chen et al. [13] estimated the release of microplastics from 18 brands of medical masks into the water (shaking at 120 rpm, 24 h). In this way, from 159.80 ± 46.14 to 222.17 ± 98.79 particles/medical mask was released when the masks were new, but the numbers increased significantly when the masks were already used (1146.00 ± 307.60 to 1478.00 ± 265.80 particles/mask). Both fragments and fibres were found in water. The second study by Wang et al. [14] incubated the three layers of the medical mask separately in water with added quartz sand (shaking at 300 rpm, 24 h). The authors report that the release of microplastics depends on the UV weathering of the masks, addition of the sand as well as on the layer of the mask. They estimated that around 483,888 plastic particles could be released from one virgin disposable mask, and 1,566,560 particles from the weathered mask if the whole mask would disintegrate.

Over the past 15 years, tremendous research efforts have been made to understand the global occurrence, distribution and potential environmental hazards of microplastics and their associated chemicals [30, 31]. Research on the potential adverse effects of microplastics on organisms has been very intense over the past decade which has demonstrated physiological perturbations in organisms exposed to microplastics (e.g., alterations to behaviour, immune responses, energy allocation, life traits), and potential links to altered ecosystem function (for reviews see [32–34]).

The aim of this paper was to thoroughly characterise the microplastics obtained from commercially available polypropylene medical masks and to evaluate their acute toxicity to the planktonic crustacean *Daphnia magna*. The microplastics from the inner frontal, middle filtering, and outer layers of disposable medical masks were investigated separately in terms of their size, shape, organic chemical leaching and aquatic toxicity. We discuss the properties of polypropylene microplastics from medical masks in line with the literature reports on plastics-associated chemicals in other polypropylene products. We provide an extensive overview of currently available ecotoxicity data for microplastics from other polypropylene-based products, as currently very limited data for microplastics from medical masks exist. Finally, we identify the knowledge gaps to guide further research in the field.

## Methods

### Milling of medical masks to produce microplastics

We produced microplastics from exemplary medical masks (sold as polypropylene, with three layers, the outer layer was blue) which were obtained from local supplier of medical protective equipment. The three layers were milled separately according to our established protocols [35]. Briefly, the material of each layer was separately cut with scissors into small pieces (~ 0.5 cm^2^), which were placed in a milling bowl. This milling bowl was put into liquid nitrogen, and left frozen for 4 min. Then the samples were milled, following a ‘quasi-cryo-milling’ procedure, whereby instead using liquid nitrogen as the coolant for the cryo-milling, the liquid nitrogen was used to maintain the material frozen before milling. This was carried out with a horizontal homogeniser (Milli Mix 20; Domel, Slovenia) with milling balls (diameter, 25 mm). The milling of the samples was performed at a horizontal frequency of 28 Hz, for 2.5 min. After milling, the samples were sieved through a 250-μm-pore sieve.

### Characterisation of microplastics

#### Fourier-transform infrared spectroscopy

To determine the main material used in the three layers of the medical masks, attenuated total reflection–Fourier-transform infrared spectroscopy (diamond crystal) was carried out (FTIR Spectrum Two spectrometer; PerkinElmer). The spectra were recorded from 400 cm^− 1^ to 4000 cm^− 1^ with an average of four scans at 4 cm^− 1^ resolution (Supplementary information Fig. S1).

#### Size and shape analysis

The particles were characterised in terms of size, shape and chemical composition. The shape of microplastics as well as the structure of intact mask layers was characterized using a field emission scanning electron microscope (FE-SEM, Zeiss ULTRA plus, Carl Zeiss, Germany). The samples were sputtered before observation with a 10 nm thin layer of Au/Pd coating. Microscopy was performed at 5.3 mm working distance using a secondary electron detector, 2 kV accelerating voltage and 30 μm aperture size. The particle size distribution was determined using a particle sizer Microtrac Bluewave as described in Selonen et al. [35]. The samples were measured in three sequentially performed runs, from which averages were calculated and used in the data analysis. The diameters of the fibres in intact mask layers were evaluated using imageJ software on SEM images (n_data_ = 60 for each of the layer).

#### Gas chromatography–mass spectrometry analysis

For each of the inner frontal, middle filtering and outer layers of the medical mask, 0.12 g of the milled material was weighed into glass vials with PTFE lid and 0.7 g methanol was added. A blank with methanol only was also prepared. The exact mass of the methanol was recorded. The vials were sealed, put in an autoclave and heated to 100 °C for 144 h. After cooling, the vials were removed and centrifuged at 9000 rpm. A small amount of methanol solution was transferred into 0.2-mL GC-MS vials using a syringe. A drop of methanol spiked with diethyl adipate as internal standard was added to each, and the vials were sealed. The exact mass of the spiked methanol was also recorded.

Gas chromatography–mass spectrometry analysis was performed on a 7890B gas chromatograph (Agilent, Santa Clara, CA, USA) coupled with a quadrupole mass detector (5977B). The GC-MS conditions were as follows: column, DB-5 MS Ultra Inert (Agilent, Santa Clara, CA, USA); injected volume, 1 μL; inlet temperature, 250 °C; carrier gas, He; and split ratio, 1:20. Temperature program: Initial temperature, 45 °C; hold time, 5 min; ramp rate, 10 °C/min; final temperature, 300 °C; hold time, 10 min. The components were identified based on the mass spectra in comparison with probability-based matching (Agilent, Santa Clara, CA, USA). A total number of peaks with an initial area > 30,000 and initial threshold > 15.0 were counted. Quantitative analysis was performed based on the peak area of each component compared to the peak area of the internal standard. An area of the particular peak from the procedural blank was subtracted. A relative response factor of 1 was used in the calculations.

### Toxicity of medical mask microplastics to *Daphnia magna*

Acute toxicity tests were performed according to the ISO 6341:2012 [36]. The daphnids used were derived from Daphtoxkit F™ *magna*. Hatched daphnids less than 24 h old were fed with algae (*Desmodesmus subspicatus*) for 1.5 h (density 5 × 10^4^ cells/mL) prior to the microplastics exposure. The microplastics from the milled layers of the medical mask were added to the ISO 6341:2012 test medium (11.76 g CaCl_2_ 2H_2_O, 4.93 g MgSO_4_ 7H_2_O, 2.59 g NaHCO_3_, 0.23 g KCl, dissolved to 1 L in water) with 0.0024% (v/v) Tween40, and the suspensions were stirred with vortex prior to dilutions and pipetting. For each test concentration 4 Petri dish with 10 mL test medium were prepared, and 5 neonates were placed into each of the Petri dish. Controls that contained only the test medium and test medium with 0.0024% (v/v) Tween40 were included in all of the experiments. The daphnids were exposed for 48 h at 21 ± 1 °C under a 16:8 h light/dark regime. The exposure during the toxicity testing was static, without mixing of the test suspensions. Three concentrations were tested: 1 mg L^− 1^, 10 mg L^− 1^ and 100 mg L^− 1^. Two separate experiments were carried out, with 20 daphnids per microplastic and control each time. After 24 h and 48 h of exposure, the daphnids were inspected for mobility (according to ISO 6341:2012 [36]), and their mortality was determined by the absence of a heartbeat.

### A review of ecotoxicity data on polypropylene microplastics

The literature search was carried out in September 2021. Two databases: Web of Science and ScienceDirect were searched using two keyword combinations: “microplastics” AND “polypropylene”; and “microplastics” AND “polypropylene” AND “toxic*”. In both knowledge bases only research articles within Environmental Sciences category were considered. Preliminary title and abstract screening were used to exclude irrelevant literature. In the second stage, the selected papers were inspected in detail for relevance to the review.

## Results and discussion

### Characteristics of microplastics obtained from medical masks

#### Polymer chemistry

The spectra from the Fourier-transform infrared spectroscopy of the inner frontal, middle filtering and outer layers of the mask materials had absorption bands at the same positions and with the same relative intensities as spectra obtained for polypropylene, from the internal database of the Slovenian National Building and Civil Engineering Institute (Supplementary Information Fig. S1). This confirmed that the source mask indeed contained polypropylene.

#### Size and shape of microplastics

**Fig. 1:**
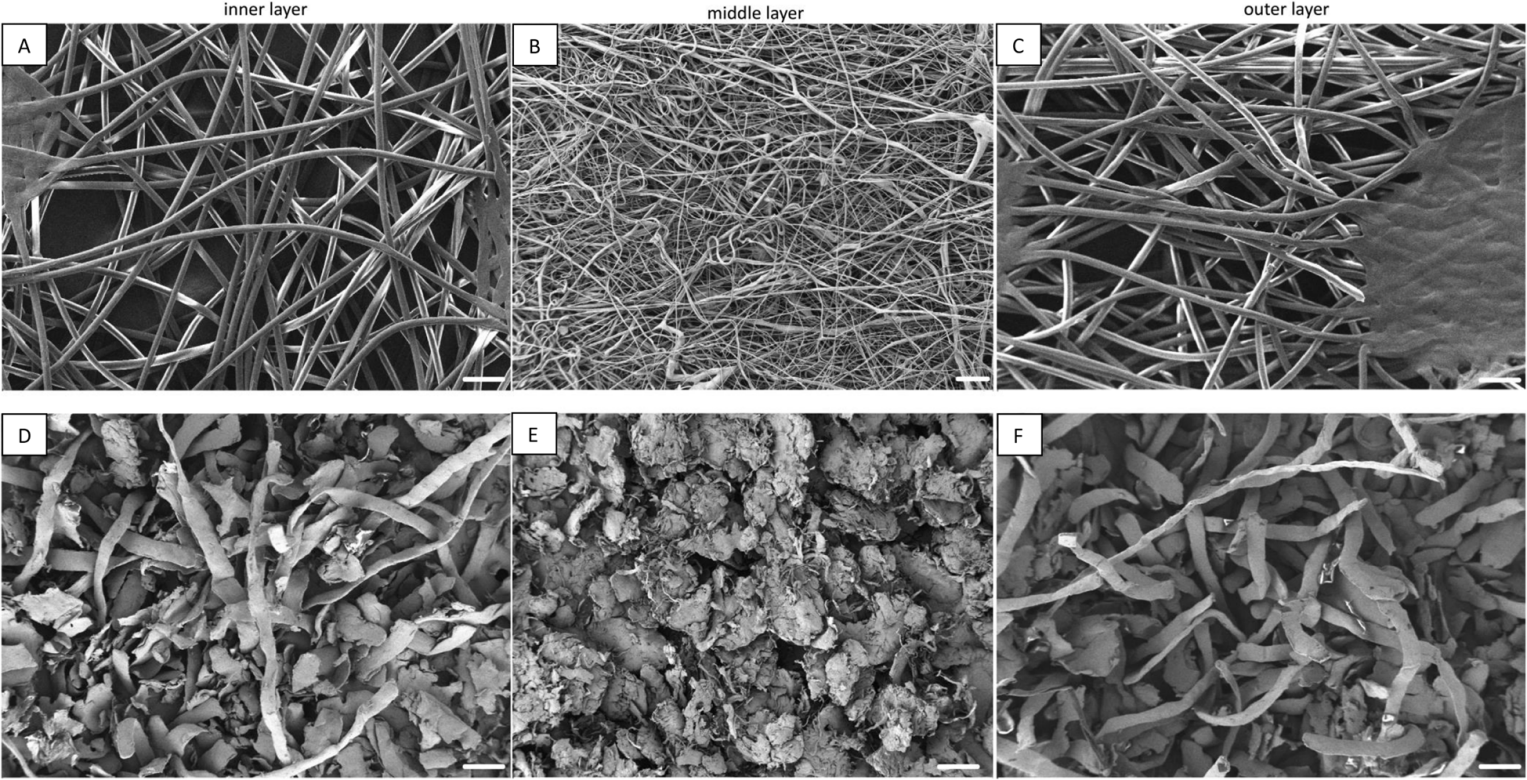
Representative scanning electron microscopy images of the intact mask layers (**A-C**) and milled microplastics (**D-F**) derived from the medical mask inner frontal layer, middle filtering layer and outer layer. White bars on the images represent 100 μm

Two different shapes of microplastics were obtained from the three layers: fibres resulted from milling of the inner and outer layers, while milling of middle layer resulted in irregularly-shaped fragments (FE-SEM Zeiss Ultra Plus; Fig. 1 D-F). This is not surprising given the fact that the composition of the intact source medical mask material was different between the layers: the inner layer and outer layer were very similar in shape being composed of a mesh of fibres with very uniform diameters (21.2 ± 1.5 μm and 22.1 ± 1.6 μm for inner and outer layer, respectively), while the fibres in the middle layer were significantly thinner (4.3 ± 2.2 μm). The fibres in middle layer had various diameters and the mesh was more compact and interwoven (Fig. 1 A-C). This difference is in line with the fact that these layers are produced using different technological approaches (see introduction and ref. (9)). Also, Ellison et al. [10] reported that thinner fibres are formed by melt-blown process used for the middle layer than by the spun-bond processes used for the inner and outer layers.

We observed that cryo-milling deforms the shape of the original fibres in medical mask. It is unclear how relevant these particles are for ecotoxicity testing in comparison to those released in the environment. Wang et al. [14] reported that different shapes of microplastics, mostly fragments of fibres, were released from the three layers of medical masks after UV weathering. Interestingly, the middle layer was more susceptible to UV than the inner and outer layers. Extraction of fibre fragments from the water after aging could be an option to obtain relevant testing materials, but the recovery in this case is low and would not be sufficient for large scale experiments or for soil toxicity testing where large quantities are needed. Therefore cryo-milling remains the most common approach to produce microplastics for research as it enables sufficient amount of testing material to be produced. Other approaches that had been used previously to produce microplastics from larger plastic items all include some mechanical fragmentation, these are: cutting with scissors, grinding with mortar and liquid nitrogen, and cutting with cryogenic microtome (Table 1).

**Table 1:**
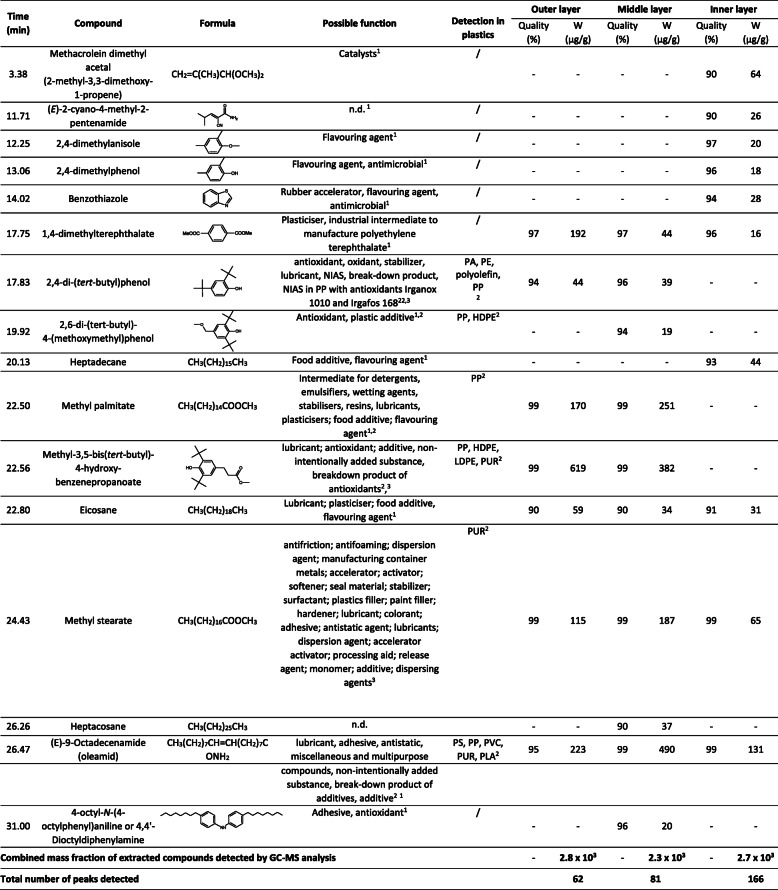
Results of the GC-MS analysis for the three layers of the disposable medical mask, with total numbers of peaks detected, combined mass fraction of extracted compounds detected and a list of compounds with ≥90% quality and at levels of > 10 μg/g microplastics. The possible functions of chemicals were extracted from Zimmermann et al. [37] and Groh et al. [38] through the database “Chemicals associated with plastic packaging”. For the chemicals which were not listed in any of these two publications, the function was summarised from the PubChem database. Where available, detection in other plastic samples was described (after Zimmermann et al. [37])

^1^ derived from https://pubchem.ncbi.nlm.nih.gov, ^2^ Zimmermann et al. [37], ^3^Groh et al. [38]; n.d. could not find the function in plastic production, PP- polypropylene, PVC- polyvinyl chloride, PUR- polyurethane, HDPE- high density polyethylene.

**Fig. 2:**
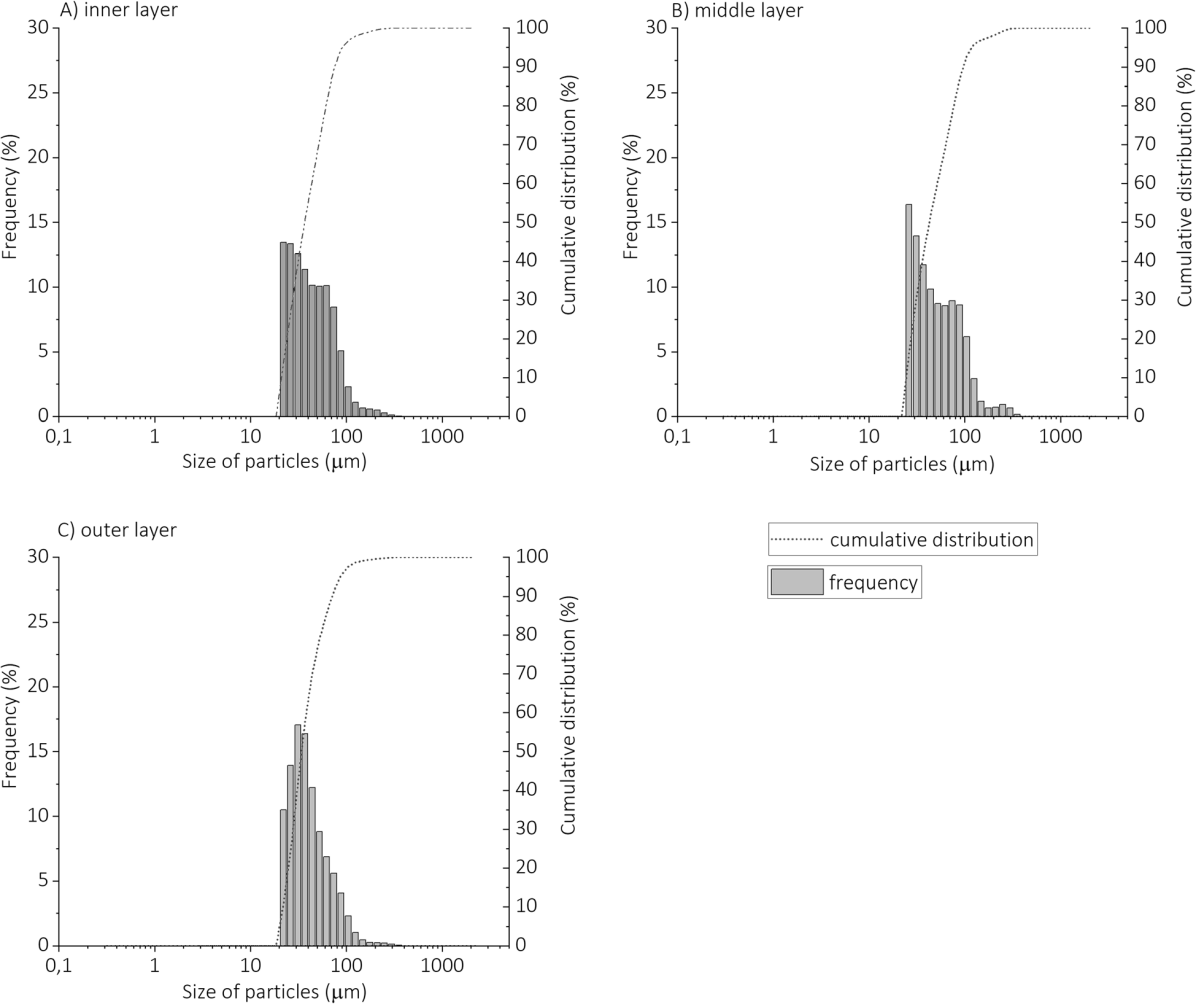
Numerical particle size distributions of the milled microplastics derived from the medical mask inner frontal layer (**A**), middle filtering layer (**B**) and outer layer (**C**), as determined by laser diffraction analysis

The particle size distributions obtained by laser diffraction analysis (Microtrac S3500 Bluewave) were very similar for the inner and outer layers, with mean sizes (±standard deviation; expressed as the equivalent diameters of spherical particles) of the fibres of 45.1 ± 21.5 μm and 42.0 ± 17.8 μm, respectively. The fragments of the middle filtering layer were slightly larger, at 55.6 ± 28.5 μm. As can be seen from the size distributions shown in Fig. 2, for the inner, middle and outer layers, 99.1%, 97.6% and 99.4% of the particles, respectively, were < 176 μm, which is not too surprising given that they were sieved through a 250-μm fine-mesh sieve.

Wang et al. [14] reported that the particle size distributions differed between the layers of medical mask weathered in water. The particles from the outer layer were mainly distributed in the range of 20–100 μm and 100–500 μm, particles from the inner layer were mainly distributed in 30–100 μm and 100–500 μm, and for the middle layer, the particle size of the microplastics was 50–200 μm. Most of the particles released were less than 200 μm in size for all three mask layers, with this trend being particularly pronounced for the middle layer, where this size distribution accounted for 91.2% of the total concentration. This means that the size range of particles obtained by cryo-milling in our case study is within the environmentally relevant values, although the particle sizes will largely depend on the choice of the parameters used for the milling method as well as for sieving.

#### Analysis of plastics-associated chemicals

We analysed extracts from the milled microplastics from the inner frontal, middle filtering and outer layers of the medical mask. GC-MS chromatograms solvent (methanol), procedural blank and of the extracts are presented in Figs. S2-S6 (Supplementary information). The data for the different compounds identified from the three layers of the medical mask are given in Table 1. This revealed several long-chain hydrocarbons; however, many of these are not listed in Table 1 because the identification of long-chain hydrocarbons is not reliable.

Among most common groups of chemicals were: antioxidants, such as 2,4-di-(*tert*-butyl) phenol; 2,6*-di*-(*tert*-butyl)-4-(methoxymethyl)phenol and methyl-3,5-bis (1,1-dimethylethyl)-4-hydroxy-benzene-propanoate (also known as Metilox); and lubricants: e.g. methyl palmitate; methyl-3,5-bis (1,1-dimethylethyl)-4-hydroxy-benzene-propanoat;, eicosane; methyl stearate and (E)-9-octadecenamide (i.e., oleamide). Some oleamide was detected in the procedural blank as well (Supplementary Fig. S3). Of particular interest, some of the compounds detected from the extraction of the inner layer are commonly used as food flavourings and antimicrobial agents (e.g. 2,4-dimethylanisole; 2,4-dimethylphenol; benzothiazole; heptadecane). The total amounts of the extracted compounds detected by GC-MS were similar for each of the layers of the medical masks (Table 1), but there were indications of many more compounds in the GC-MS chromatogram from the extraction of the inner frontal layer (Supplementary Fig. S2).

It has been reported that a number of different compounds can leach from polypropylene products [39]. For example, a total of 107 analytes were identified in leachates from polypropylene food containers [39]. Among these, the most abundant groups were antioxidants and their degradation products (tris (2,4-di-tert-buthylphenyl)phosphite; 2,6-Di-tert-butyl-4-ethyl-phenol), plasticizers (e.g. bis-(2-ethylhexyl) phthalate; dibutyl phthalate), cross-linking agents (e.g. 2-mercaptobenzothiazole; benzothiazole) and other additives (e.g., antistatic agents; lubricants; non-ionic surfactants) [39]. Similarly, Zimmermann et al. [37] reported a number of chemicals in PP products, for example 18, 5 and 22 different chemicals in gummy candy packaging, handkerchief packaging, and shampoo bottle, respectively. Some of these chemicals were also detected in the medical masks characterised in this study (Table 1). We could not find data specific to medical masks, but there are some records that medical masks might contain formaldehyde and bromo-2-nitropropane-1,3-diol (bronopol), which can cause acute dermatitis in healthcare workers [40], but these were not identified in the present samples.

### Toxicity of polypropylene microplastics to *Daphnia magna*

**Fig. 3:**
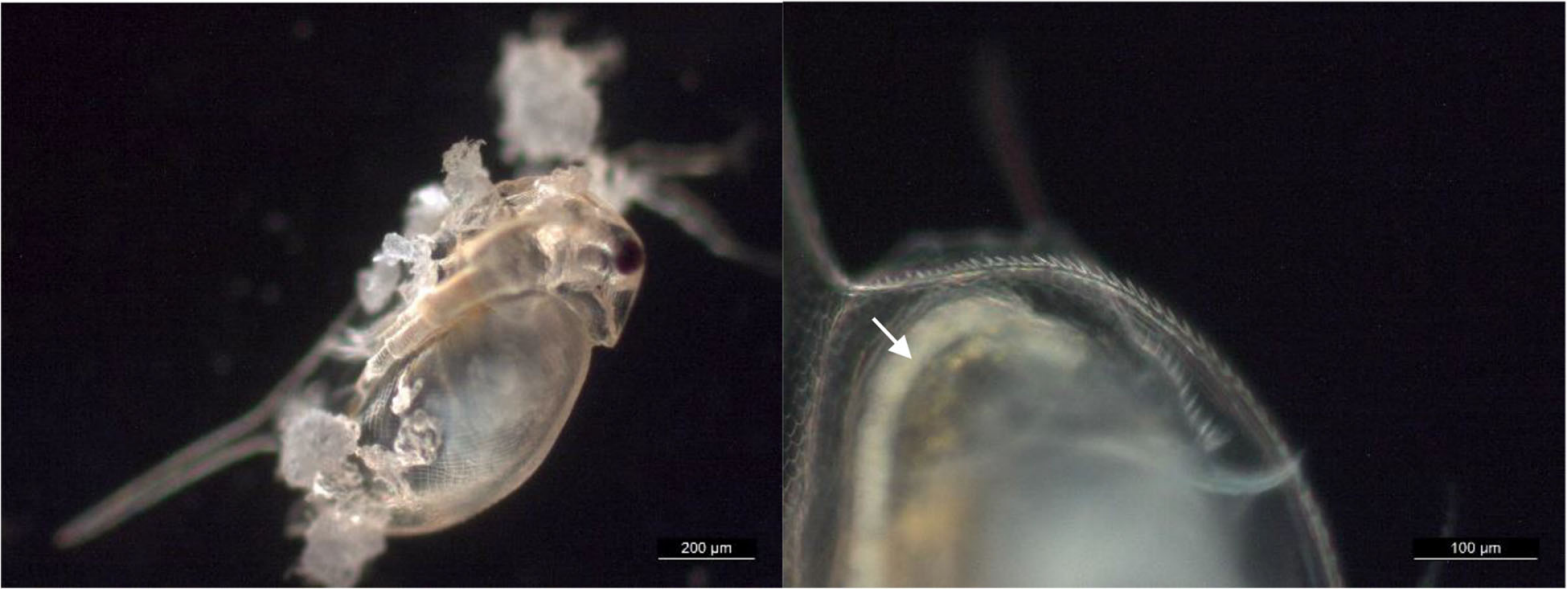
Representative light microscopy images of *Daphnia magna* after 48 h exposure to the medical mask microplastics. Left: Microplastics from the middle filtering layer of the medical mask attached to the body surface of a *D. magna*. Right: Microplastics from the inner frontal layer of the medical mask in the gut of a *D. magna* (white arrow)

We observed no effects on the mobility and survival of *D. magna* exposed to the three types of microplastics that were milled from the three layers of the medical mask at 1 mg L^− 1^-100 mg L^− 1^ for 48 h. However, there was attachment of these microplastics to the body surface and ingestion of the microplastics by *D. magna* (Fig. 3). This is in line with our previous work where no acute effects of polyethylene cosmetic beads and polyester textile fibres on *D. magna* were recorded, but these microplastics were as well found in the gut [41, 42]. Acute effects were however observed in the case of polyester textile fibres when the exposure was prolonged for additional 24 h as the daphnids could not recover from the exposure [41]. We would thus suggest the need for further studies on chronic effects of microplastics from medical masks. Of note, chronic studies have already shown numerous effects of other types of microplastics on *D. magna* [43, 44]. Furthermore, multigenerational studies with daphnids have shown that some effects, such as decreased reproduction, can persist over at least two generations without further exposure to the microplastics [43]. The choice of exposure scenarios and endpoint selection in future polypropylene microplastics ecotoxicity studies with *D. magna* should also consider the physicochemical properties that appear particular for this type of test material [45], as well as testing for the expected chemically and physically induced interactions of microplastics with the test organisms, for example adsorption onto the body surface and interference with moult.

Although several plastics-associated chemicals were identified in methanol extracts from medical mask microplastics (Table 1), obviously the concentrations in test medium during the acute exposure of *D. magna* were not high enough to cause acute lethal effects. Similarly, when leachates from 26 different plastic products were tested (analysed at 100–250 g plastics L^− 1^ water), none of the leachates from polypropylene were toxic to the water flea *D. magna* [46]. Also, in another study, leachates from polypropylene showed the lowest inhibition of the survival and settlement of the barnacle *Amphibalanus amphitrite* when compared to high-density and low-density polyethylene, polyvinylchloride, polycarbonate, polyethylene terephthalate, polystyrene (all analysed at 1000–5000 cm^2^ L^− 1^ with water; equivalent to 100–500 g plastics L^− 1^ water) [47].

Currently, there is only one very recent ecotoxicity study available on the polypropylene microplastics from medical masks [48]. The authors report the effect of microplastics obtained from FFP2 medical mask on springtails *Folsomia candida* and earthworms *Eisenia andrei*. The reproduction and growth of juvenile springtails and spermatogenesis of earthworm were decreased already at environmentally relevant concentration (Table 2). No induction of oxidative stress and effects on survival were found for both species. To our knowledge, no data on the effects of medical mask microplastics for aquatic organisms currently exists.

**Table 2:** Overview of the ecotoxicity studies on microplastics derived from polypropylene

Species	MPs					Exposure time	Effects	Ref.
	Form	Dimension (μm)	Source material	Mode of preparation	Test concentrations			
*amphipod Hyalella Azteca (Crustacea)*	Fibres	Diameter: 20 Length: 20–75	Aged marine fishing rope (3 years under ambient conditions)	Cutting the rope by scissors	0–90 fibres mL^− 1^	10 days	↑ Mortality, ↓ Growth, ↓Weight; ↑ egestion time; Fibres not retained in the gut LOEC = 45 fibres mL^− 1^	[49]
*shrimp Palaemonetes pugio (Crustacea)*	Fibres	a) Diameter: 30–38 b) Diameter:80–105 Length: not defined	Aged marine fishing rope (3 years under ambient conditions)	Cutting the rope by scissors	50,000 fibres L^−1^	96 h	a) no effect b) ↑ Mortality	[50]
*shrimp Palaemonetes pugio (Crustacea)*	Fragments, irregular shapes	30–38 80–105	Purchased, TWOH Chem	Sieving the powder	50,000 particles L^− 1^	96 h	No effect	[50]
*shrimp Litopenaeus vannamei (Crustacea)*	Fragments, spherical, rod-shaped, sheet-like	1.77–18	Purchased, China Petroleum & Chemical Corporation	Used as received	1 mg L^−1^	14 days	Changes in gut microbial composition ↑ proteome expression related to moult processes and metabolism Changed metabolic profile No effect on immune related proteome expression	[51]
*decapod Nephrops norvegicus (Crustacea)*	Fibres	Diameter: 200 Length: 3000–5000	Fishing rope	Fibres were removed from twisted split rope	Adding 5 fibres every two days. Total fibres at the end: 360	8 months	↓ Feeding rate; ↓ Body mass ↓ Metabolic rate Retention of fibres in foregut	[52]
*mole crab Emerita analoga (Crustacea)*	Fibres	Diameter: 100 Length: 1000	Fishing rope	Cutting the rope by scissors	3 fibres L^−1^	72 days	↑ Mortality ↓ Reproductive success	[53]
*nematode Caenorhabditis elegans (Annelida)*	Fragments, irregular shapes	< 70	Purchased, Sigma-Aldrich	Ground with mortar, sieved < 200 μm	0.5–5.0 mg m^−2^ (agar plate)	48 h	↑ Mortality; ↓ growth; ↓ Reproductive success; ↑ stress genes LOEC = 0.5 mg m^− 2^	[54]
*nematode Caenorhabditis elegans (Annelida)*	Fragments, irregular shapes	Three size ranges: a) < 250, b) 250–630, c) 630–1000	Obtained from Bundesanstalt für Materialforschung und prüfung (Berlin, Germany)	Cryo-milling; sieving < 1000 μm	0.01, 0.1 and 1% w/w soil	24 h	a) ↓ reproduction at 1% b, c) no effect	[55]
*ragworm Hediste diversicolor (Annelida)*	Fragments, irregular shapes	< 400	Purchased, supplier not provided	Cryo-milling; sieving < 400 μm	1 and 5% w/w sediment	10 days	↓ coelomocytes viability, ↓ phenoloxidase, ↓ acid phosphatase, no effect on phagocytic activity; LOEC = 10 mg kg^− 1^	[56]
*oyster Crassostrea gigas (Mollusca)*	Fragments, irregular shapes	< 400	Purchased, supplier not provided	Cryo-milling; sieving < 400 μm	0.008, 10, 100 μg L^− 1^	10 days	No effects on clearance rate of organisms, tissue alteration, antioxidant defence, immune response and DNA damage.	[57]
*mussel Mytilus spp (Mollusca)*	Fragments, irregular shapes	< 400	Purchased, supplier not provided	Cryo-milling; sieving < 400 μm	1 and 1000 mg L^− 1^	10 days	↑ antioxidant response No effect on the clearance rate, and histopathological parameters	[58]
*mussel Perna viridis (Mollusca)*	Fragments, irregular shapes	< 30 30–300 300–1000	Provided by the Faculty of Engineering and Industrial Technology, Silpakorn University, Thailand	Used as received	66, 333, 666, and 1333 particles L^−1^	96 h	Total mortality after 96 h, After 72 h 67%, 63% and 70% mortality for the small, medium and large particles, respectively. About 90% of the available MPs were rejected as pseudofaeces by the mussels, with approximately 10% of MPs being ingested and accumuled in the soft tissue.	[59]
*clam Donax trunculus (Mollusca)*	Fragments, irregular shapes	100–400	Purchased, supplier not provided, mixture of pellets from PP and PE	Cryo-milling; sieving < 400 μm	0.06 g kg^− 1^ of sand	3 h, 1, 2, 3, 4,7,10 and 15 days	↑ oxidative stress ↓ acetylcholinesterase activity	[60]
*barnacle Amphibalanus amphitrite (Crustacea)*	a) plastic square b) leachate from plastic	a) 4 cm^2^ b) 0.50 m^2^ plastic in 1 L seawater	Storage Container	a) Cutting b) Soaking for 24 h	b) 0.1 and 0.5 m^2^ L^− 1^	24 h, 48 h, 96 h	a) 24 h–96 h: ↑ Mortality; ↓ Settlement b) 24 h: ↑ Mortality; ↓ Settlement	[47]
*microalgae Spirulina sp*	Fibres	Diameter: 15 Length: 1000	Purchased fibres	Cutting	300, 500, 550 mg L^−1^	112 days	↓ growth LOEC = 300 mg L^− 1^	[61]
*algae Chlamydomonas reinhardtii (Algae)*	Fragments, irregular shapes	< 400–1000	Disposable cup lid	Cutting to 1 cm, cryo-milling; sieving < 400 μm	1000 mg L^− 1^	72 days	No effect on growth up to 60 days, ↓ growth after 72 days ↑ stress genes, e.g. polysaccharide biosynthesis ↑ formation of polypropylene-algae hetero-aggregates	[62]
*algae Chlorella pyrenoidosa; Microcystis flosaquae (Algae)*	Fragments, irregular shapes	~ 172	Purchased, Aladdin Industrial Corporation	Used as received	5, 10, 50, 100, 250, 500 mg L^− 1^	11 days	↓ chlorophyll content ↓ photosynthetic activity LOEC = 5 mg L^− 1^, no clear dose-response	[63]
*algae Chlorella sp. (Algae)*	Fragments, irregular shapes	100–300, 300–500, and 500–700	Plastic bag	Cutting to smaller particles, ground with cryogenic mill, sieving	10, 250, 500, 750, and 1000 mg L^− 1^	3 days	↓ growth, but very small rate of inhibition	[64]
*fish Danio rerio (embryo)*	undefined	undefined	Purchased, Sigma-Aldrich	undefined	1 mg L^−1^ and 10 mg L^− 1^	96 h	↑ pericardial sac area No effect total body size	[65]
*fish Danio rerio (larvae, adults)*	Fibres	Diameter 20, length: 50 ± 26 and 200 ± 90	Not reported	Cutting with cryogenic microtome	10 and 100 μg L^− 1^	48 h larvae, 21 days adults	↑intestinal damage, larger effect for long fibres ↑ oxidative stress, inflammation and lipid depletion in the larvae gut; larger effect for long fibres ↓decreased feeding Changed metabolic profile, disruption in lipid metabolism	[66]
*fish Danio rerio (adults)*	Fragments, irregular shapes	< 70	Purchased, Sigma-Aldrich	Ground with mortar, sieved < 200 μm	0.001–10.0 mg L^− 1^	10 days	↑ Mortality; LOEC = 10 mg/L intestinal damage	[54]
*fish Danio rerio (adults)*	Fragments, irregular shapes	1–15	Purchased, Huachuang plastic material Co. Ltd.	Ground with pulverizing and filtering	0.2 mg L^−1^	28 days	↑ lipid peroxidation in the gut ↓ superoxide dismutase in liver Possible oxidative stress	[67]
*fish Pimephales promelas (embryo)*	Fragments, irregular shapes	150 to 500	Purchased, ASPX company	Ground using a burmill coffee grinder, sieved 150 μm < 500 μm,	280 and 2800 particles/L or 1.43 mg L^−1^and 14.3 mg L^− 1^, respectively.	14 days	↑ body weight No effect on hatching success, survival, and length	[68]
*fish Dicentrarchus labrax (adults)*	Fragments, irregular shapes	700–1000	Purchased, Sigma-Aldrich	Ground with a cutting mill, sieving to obtain the 700–1000 μm fraction	10% w/w	60 days	No effect on growth No effect on gut histology ↑ immune-related genes	[69]
*earthworm Metaphire guillelmi (Annelida)*	Fragments, irregular shapes	13	Purchased, Huachuang Plasticizing Corporation	Used as received	0.25% w/w soil	28 days	No changes in gut microbiota	[70]
*earthworms Eisenia fetida (Annelida)*	Fragments, irregular shapes	8–125, 71–383 and 761–1660	Purchased, Huachuang Plasticizing Corporation	Grinding with liquid nitrogen	0.25% w/w soil	14 days, 28 days	↓ antioxidant enzymes activities (14 d, 28 d) ↑ DNA damage (28 d) No changes in lipid peroxidation (28 d)	[71]
*earthworms Eisenia fetida (Annelida)*	Fragments, irregular shapes	< 150	Purchased, Sigma-Aldrich	Mechanically ground, sieving < 150 μm	0.03, 0.3, 0.6, 0.9% w/w	14 days, 28 days, 42 days	↓ growth (14, 21 and 42 days); LOEC = 0.6% ↑ mortality (42 days), LOEC = 0.3% ↑ lipid peroxidation (14, 21 and 42 days); LOEC = 0.03%; ↑ antioxidant levels (14, 21 and 42 days); LOEC = 0.3%	[72]
*White worm Enchytraeus crypticus (Annelida)*	Fragments, irregular shapes	49 141 1520	Not reported	Cryo-milling, sanding, sieving	0.032, 0.1, 0.32, 0.64% w/w	64 days	No effect on reproduction	[73]
*springtails Folsomia candida (Entognatha) earthworms Eisenia Andrei (Annelida)*	Fibres and fragments	< 300	Triple-layered disposable white face masks	cut using micro-scissors, and sieved < 300 μm	0.1% w/w	28 days	↓ reproduction and growth of juveniles springtails No effect on survival, esterase activity, oxidative stress, and light avoidance behavior of adult springtails ↓esterase activity and spermatogenesis of earthworms No effect on survival and oxidative stress in earthworms	[48]
*Mealworm larvae Tenebrio molitor (Insecta)*	Fragments	2000–3000	Purchased, SINOPEC (China) and EyeIslet (China)	cut into 2–3 mm fragments	100% (fed on this material) And 50% mixed with bran	14 days	Decomposition and feeding occur only in case of 50% No effect on survival	[74]
*garden cress Lepidium sativum*	Fibres and fragments	< 125	Not reported	grinding, with liquid nitrogen, sieving < 125 μm	0.02% (w/w)	6 days, 21 days	↓biomass, No effect on reactive oxygen species formation and antioxidants content, change ration between pigments	[75]
*plant Cucurbita pepo*	Fibres and fragments	40–50	Purchased, Sigma-Aldrich	Not reported	0.02, 0.1, 0.2% w/w	28 days	↓ root and shoot growth No effect on leaf area and photosynthesis Changes in concentrations of elements in leaves	[76]

### A review of ecotoxicity data on microplastics from other polypropylene-based products

Many literature reviews have indicated that polypropylene microplastics are among the least studied microplastics in laboratory ecotoxicity studies [77–79]. This is surprising given the fact that polypropylene is the second largest European and global plastic resin in terms of production volume [80, 81] and polypropylene microplastics are among the most common found in the environment [77]. For example, of the total of 157 peer-reviewed ecotoxicity articles published by 2018 with 612 different microplastics on aquatic organisms, only 12.1% included polypropylene [33]. Our literature search using the keywords “polypropylene” and “microplastics” (September 2021) resulted in 688 hits within the category Environmental Sciences of the Web of Science knowledge base and 2003 hits within ScienceDirect. For the keyword combination “polypropylene” and “microplastics” and “toxic” the number of hits was 59 and 1175 for Web of Science (WoS) and ScienceDirect, respectively (Table S1 Supplementary information). After abstract inspection, in total 27 studies were identified as ecotoxicity studies including species being relevant for this review. An additional 3 were found in the review by De Sá et al. [33] dealing with microplastics from fishing ropes which were not identified during our search in WoS or ScienceDirect. Interestingly, 44% of studies included in our review were recently published (2021) which indicates that the number of studies on polypropylene microplastics has increased significantly (Supplementary information Table S1, Table 2).

Three types of polypropylene microplastics have been studied in terms of their shapes and sources: fibres from the cutting of fishing rope; fragments obtained from cryo-milling of different products; and purchased fragments (pellets) from polymer producing companies. A comparative analysis of the reported adverse effects across the test species for exposure concentrations and with other microplastic polymer types is very difficult, because the various studies have used a range of test materials of different dimensions (fibres: length 20–1000 μm, width 15–200 μm; fragments: diameter ~ 10–3000 μm), and according to different concentration metrics (particle mass/volume, particle number/volume, particle mass/mass sediment or soil, particle mass/surface area of agar plate) and toxicity endpoints. Also, it was not possible to find a difference between the effects of polypropylene fibres and fragments, although it was suggested previously that the shape of the microplastics has a predominant role in some adverse effects, with fibres showing greater toxicity [49, 50].

Nevertheless, it can be concluded that both polypropylene fibres and fragments have the potential to induce adverse effects on organisms at concentrations that can already be found in the environment [49, 52, 53, 55], although some studies also tested unrealistically high microplastics levels [50] (Table 2). The environmental relevance of some of the test concentrations is difficult to assess, as the measurement metrics are different from those most commonly reported in monitoring studies (i.e., particles volume^− 1^ or km^− 2^) [82]. The potentially adverse effects induced by polypropylene microplastics are similar to those induced by other types of microplastics [33], and include: their retention in the gut; decreased feeding and growth rates; changed metabolic rates and metabolic processes; changes in moult process; decreased reproduction; stress induction, oxidative stress and antioxidant responses; induction of immune responses; alteration of the gut microbiome; and (very rarely) mortality (Table 2). However, as Rochman et al. [83] emphasised, microplastics represent a diverse suite of contaminants that show a range of different molecular structures, monomer compositions, chemical additives, sizes, shapes and colours, with many of these potentially involved in their toxicity potential. It is therefore imprecise to generalise toxicity data across microplastics types, even within the same polymer group. This implies the need for new experimental data for polypropylene microplastics from medical masks as currently only such study exists [48]. In particular, such studies should be directed towards the investigation of microplastics from weathered medical masks. Weathering affects not only the surface properties of the particles, but also the release of the additives and the plastic-derived intermediates, as well as the sorption of other environmental pollutants [18, 84, 85]. This can lead to alterations to the behaviour of the microplastics and to their bioavailability to organisms (i.e., the form in which they are available for organisms to ingest), and ultimately to their hazard potential [18, 86, 87].

## Conclusions and outlook

We have presented a case study of mechanically induced formation of polypropylene microplastics from a commercially available medical mask. Different types of microplastics were obtained from the three layers of the mask, as fibres from the inner frontal and outer layers, and irregularly shaped fragments from the middle filtering layer. The shape of the obtained microplastics differed from the initial fibrous structure of the intact medical mask layers, which indicates that the material is deformed during cryo-milling. Microplastics from the three layers differed in the organic chemical composition of their leachates. The inner frontal layer that comes into contact with the face contained more additives that function as antimicrobials and flavourings, while the middle filtering and outer layers contained more antioxidants, plasticisers and lubricants. Our preliminary acute toxicity study using the standard test organism *D. magna* did not show any severe effects of these microplastics at relatively high exposure concentrations, although adsorption and ingestion of the particles by the daphnids was observed. As evident from the review on the ecotoxicity of polypropylene microplastics derived from other polypropylene products these can induce various adverse effects on organisms at environmentally relevant values. Due to the increasing use of medical protective masks we thus suggest the need for a thorough investigation into the environmental hazards and impacts of medical mask microplastics on the environment. Further chronic ecotoxicity studies and multigeneration studies with a suite of ecotoxicity test organisms are needed.

With the development and widespread use of new advanced materials for air filtration [9] also other types of microplastics and nanoplastics could be released into the environment from medical masks. The most popular advanced materials are polymer nanofibre membranes, electret membranes, and porous filters based on metal-organic frameworks [88–90]. Filtration materials can also be treated or coated with numerous antimicrobial agents, such as metal nanoparticles, organic compounds, organic acids and sodium chloride [9]. Silver nanoparticles are often added to such materials, which can release silver ions into the environment, which pose an additional hazard [91]. There are many other variations of filtering materials under investigation, and each of these might also release different types of microplastics and/or nanoplastics.

At this point, it remains unknown how great the environmental burden of improper disposal of medical masks is. However, it is certain that the production volume and use of disposable medical masks will continue to expand globally.

## Supplementary Information

**Additional file 1.**

## Data Availability

Not applicable.
