# Supplementary material for: Environmental hazard of polypropylene microplastics from disposable medical masks: acute toxicity towards Daphnia magna and current knowledge on other polypropylene microplastics
Source: Microplast nanoplast. 2022 Jan 4;2(1):1. doi: 10.1186/s43591-021-00020-0 (PMC8724753; doi:10.1186/s43591-021-00020-0)
Supplement: Supplementary file 1 — Additional file 1. [file 43591_2021_20_MOESM1_ESM.doc]

**Supplementary Information**

**Environmental hazard of polypropylene microplastics from disposable medical masks: acute toxicity towards *Daphnia magna* and current knowledge on other polypropylene microplastics**

Anita Jemec Kokalj^1^,*, Andraž Dolar^1^, Damjana Drobne^1^, Marjan Marinšek^2^, Matej Dolenec^3^, Luka Škrlep^4^, Gregor Strmljan^4^, Branka Mušič^4^, Andrijana Sever Škapin^4^

^1^University of Ljubljana, Biotechnical Faculty, Department of Biology, Večna pot 111, SI-1000 Ljubljana, Slovenia

^2^University of Ljubljana, Faculty of Chemistry and Chemical Technology, Chair of Materials and Polymer Science, Večna pot 113, SI-1000 Ljubljana, Slovenia

^3^University of Ljubljana, Faculty of Natural Sciences and Engineering, Department of Geology, Aškerčeva 12, SI-1000 Ljubljana, Slovenia

^4^Slovenian National Building and Civil Engineering Institute; Dimičeva ulica 12; SI-1000 Ljubljana, Slovenia

***Corresponding author: Anita Jemec Kokalj**

Department of Biology

Biotechnical Faculty

University of Ljubljana

Večna pot 111

SI-1000 Ljubljana, Slovenia

Email: anita.jemec@bf.uni-lj.si

***Fourier-transform infrared spectroscopy***

**Figure S1**


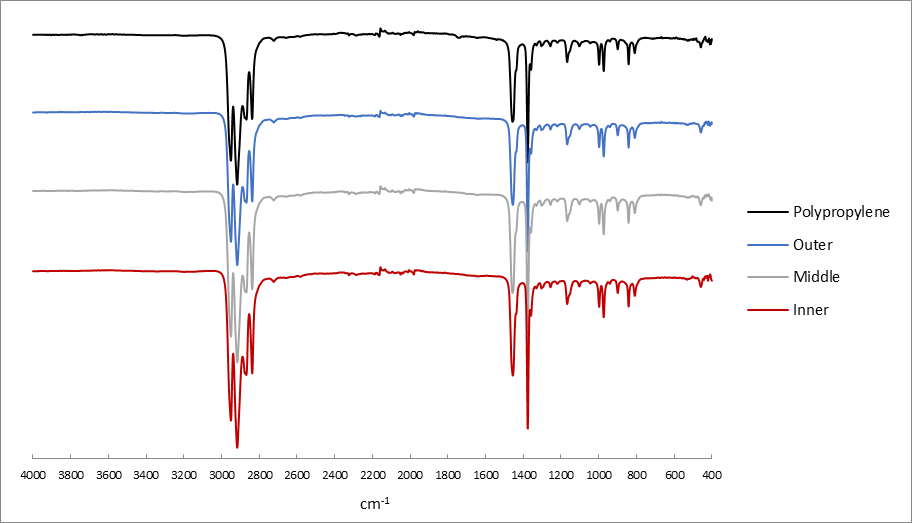


**Figure S1.** Attenuated total reflection–Fourier-transform infrared spectroscopy spectra for the polypropylene standard (from the internal Slovenian National Building and Civil Engineering Institute [ZAG] database) and for the inner frontal, middle filtering and outer layers of the milled medical mask.

***Gas chromatography–mass spectrometry analysis***

**Figures S2-S6**


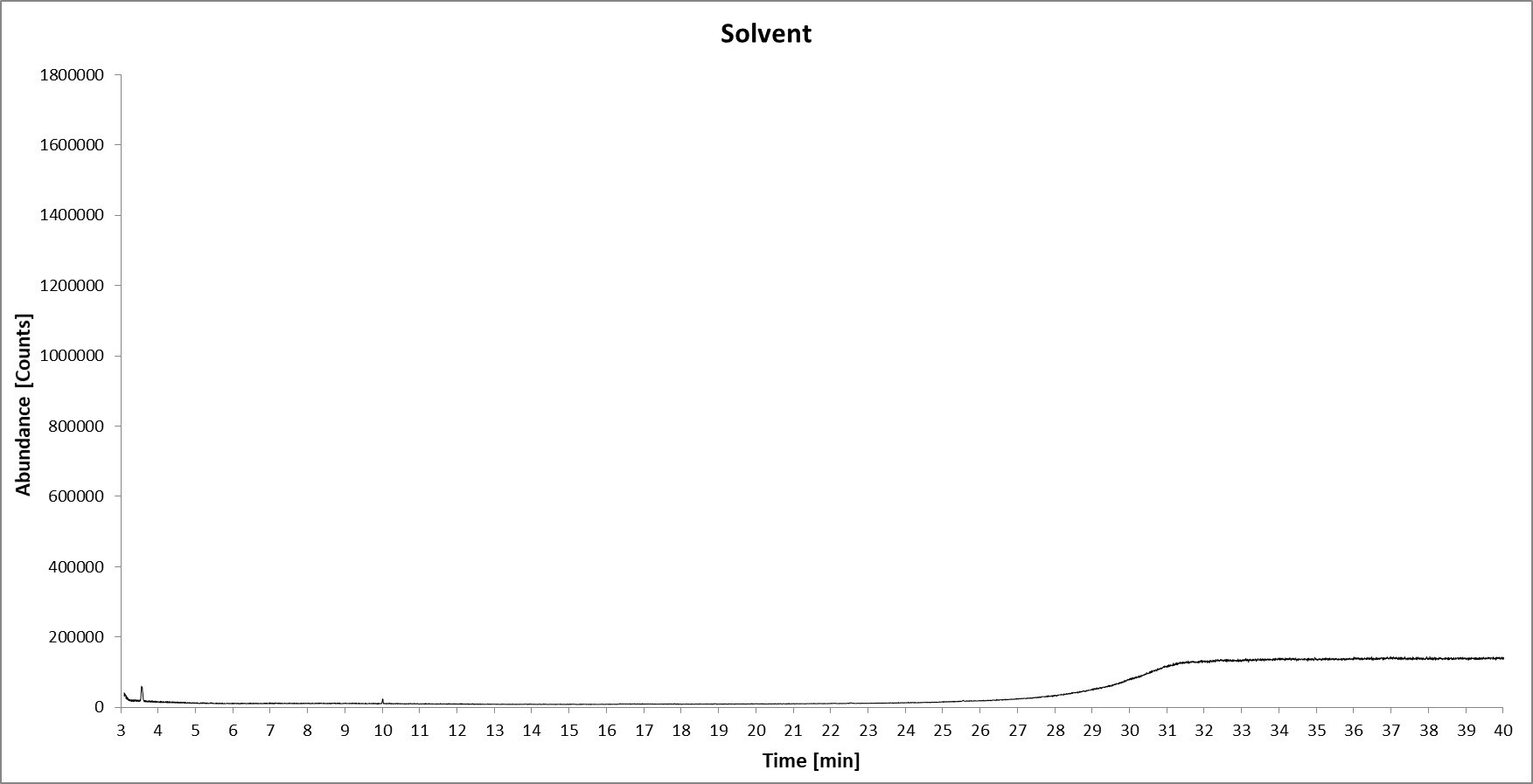


**Figure S2**. The GS-MS chromatogram of solvent (methanol).


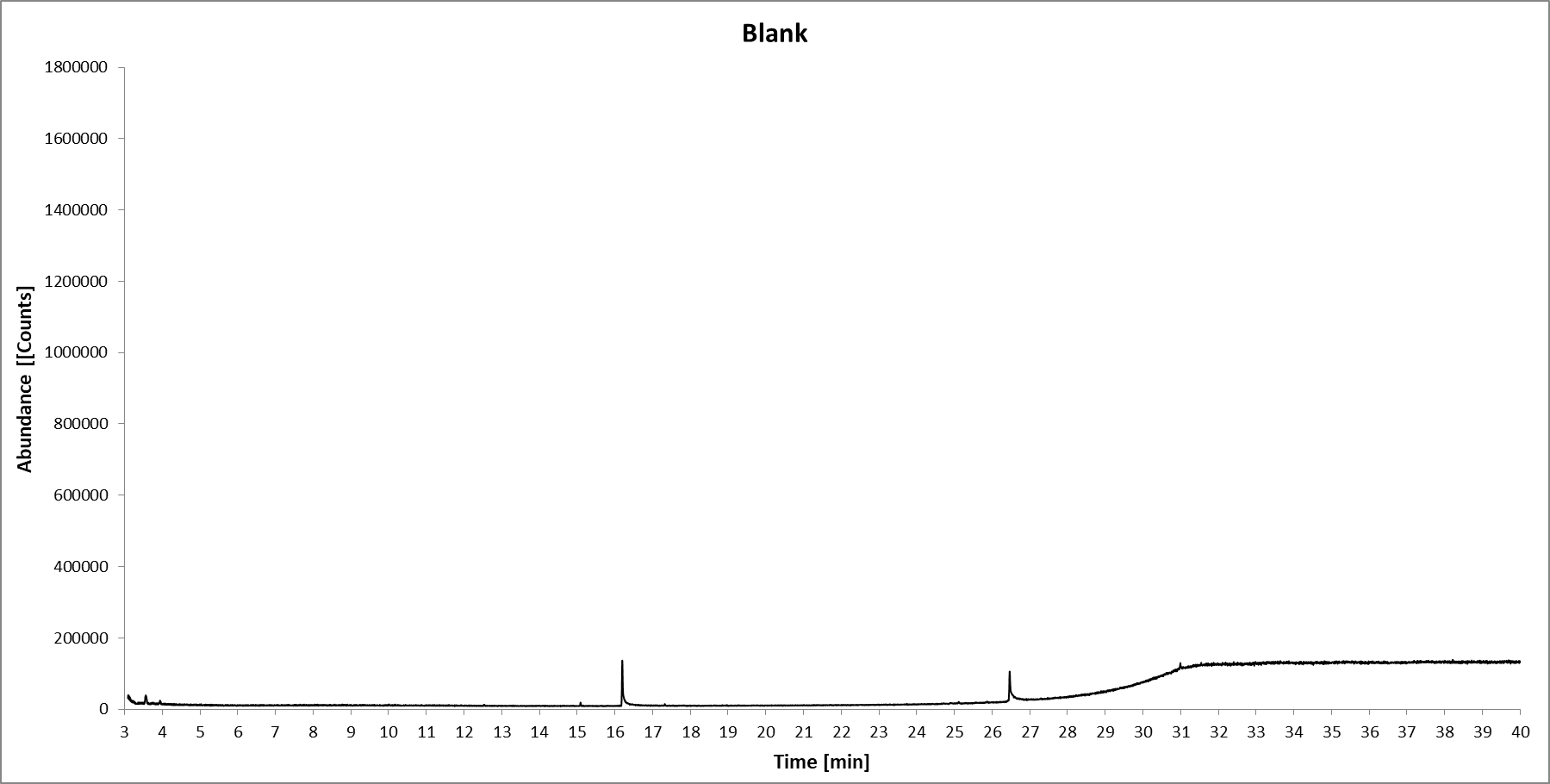
 **Figure S3**. The GS-MS chromatogram of procedural blank.


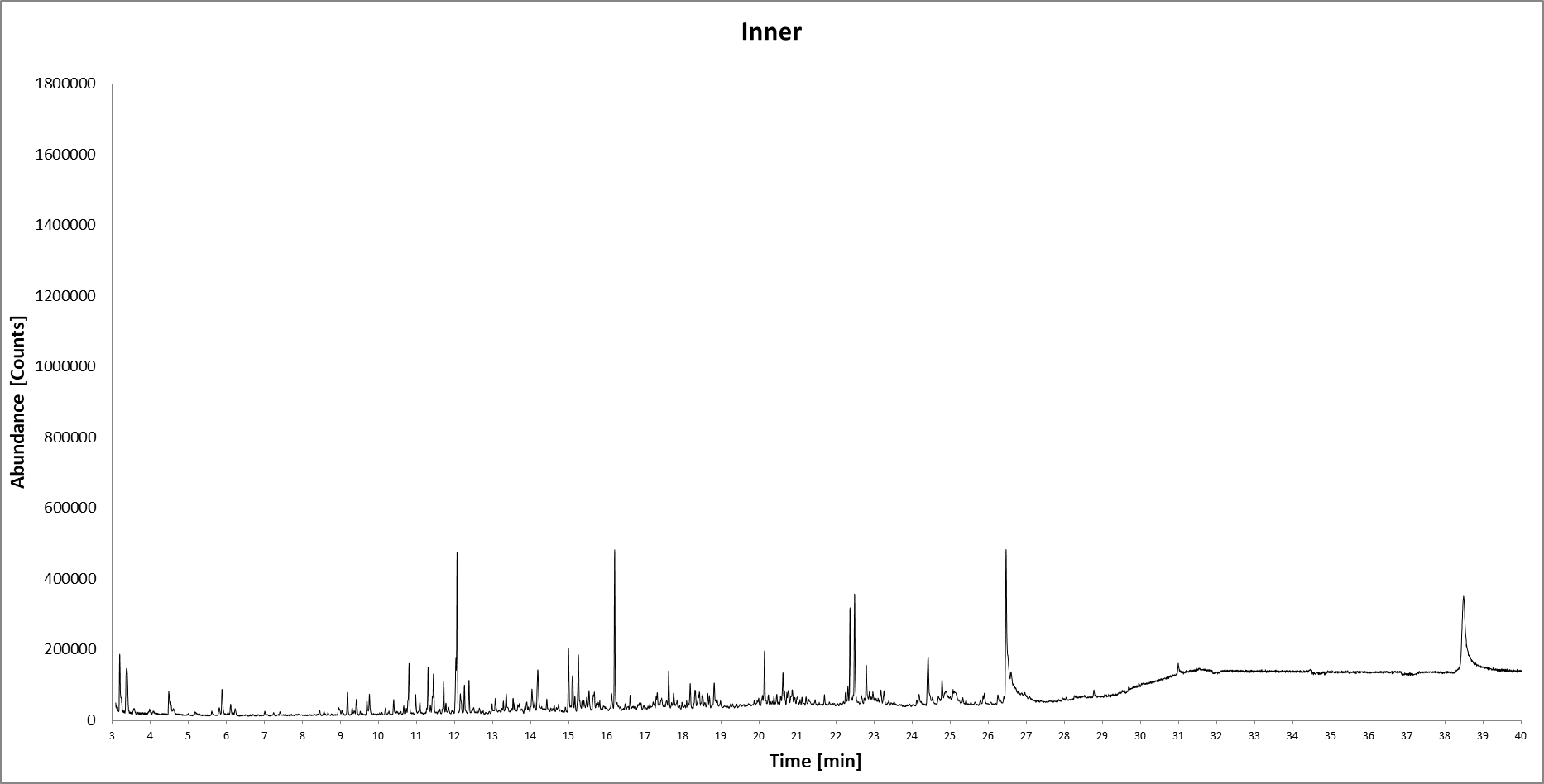


**Figure S4.** GC-MS chromatogram of the extract of the milled inner frontal layer of the face mask.


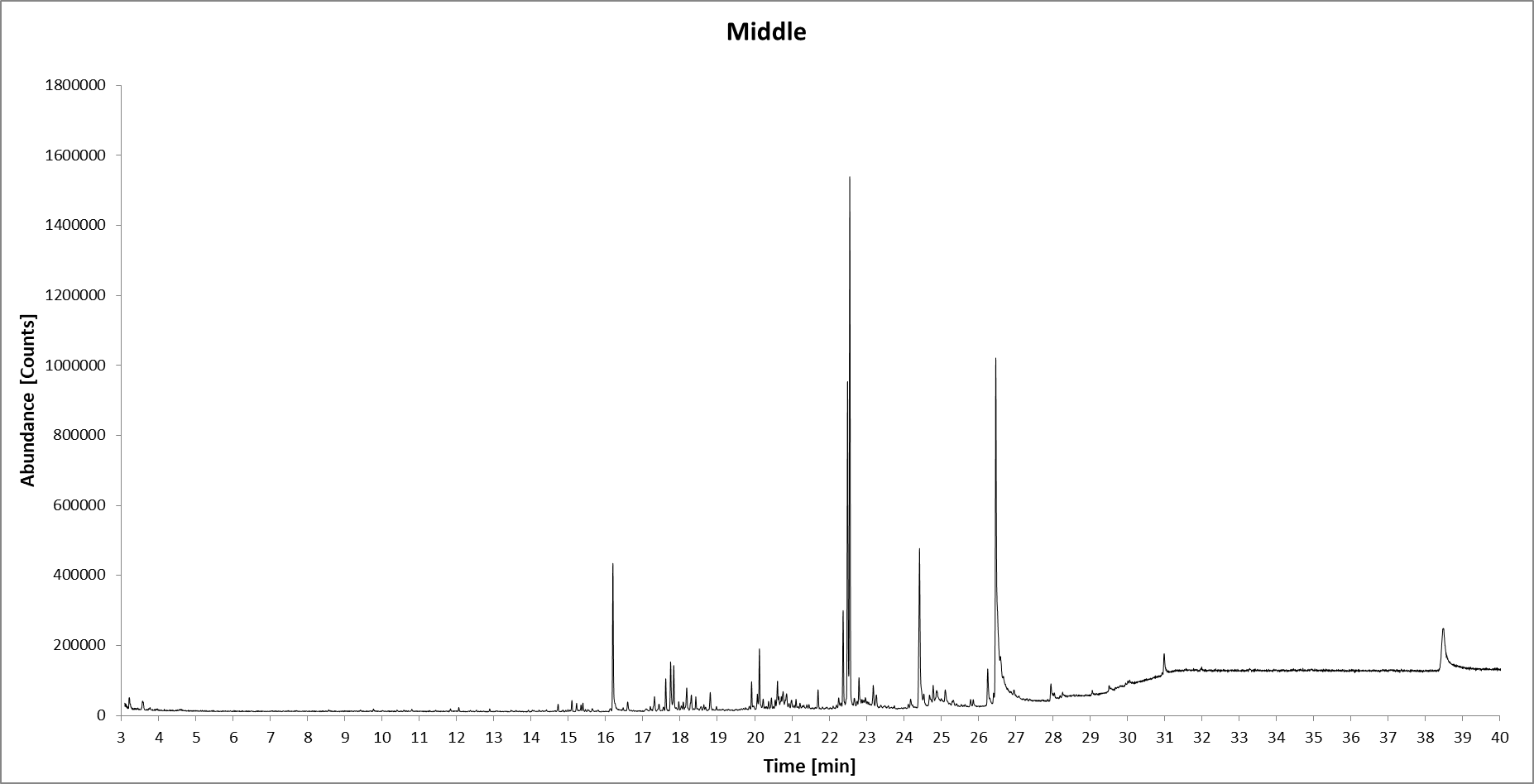


**Figure S5.** GC-MS chromatogram of extract of the milled middle filtering layer of the face mask.


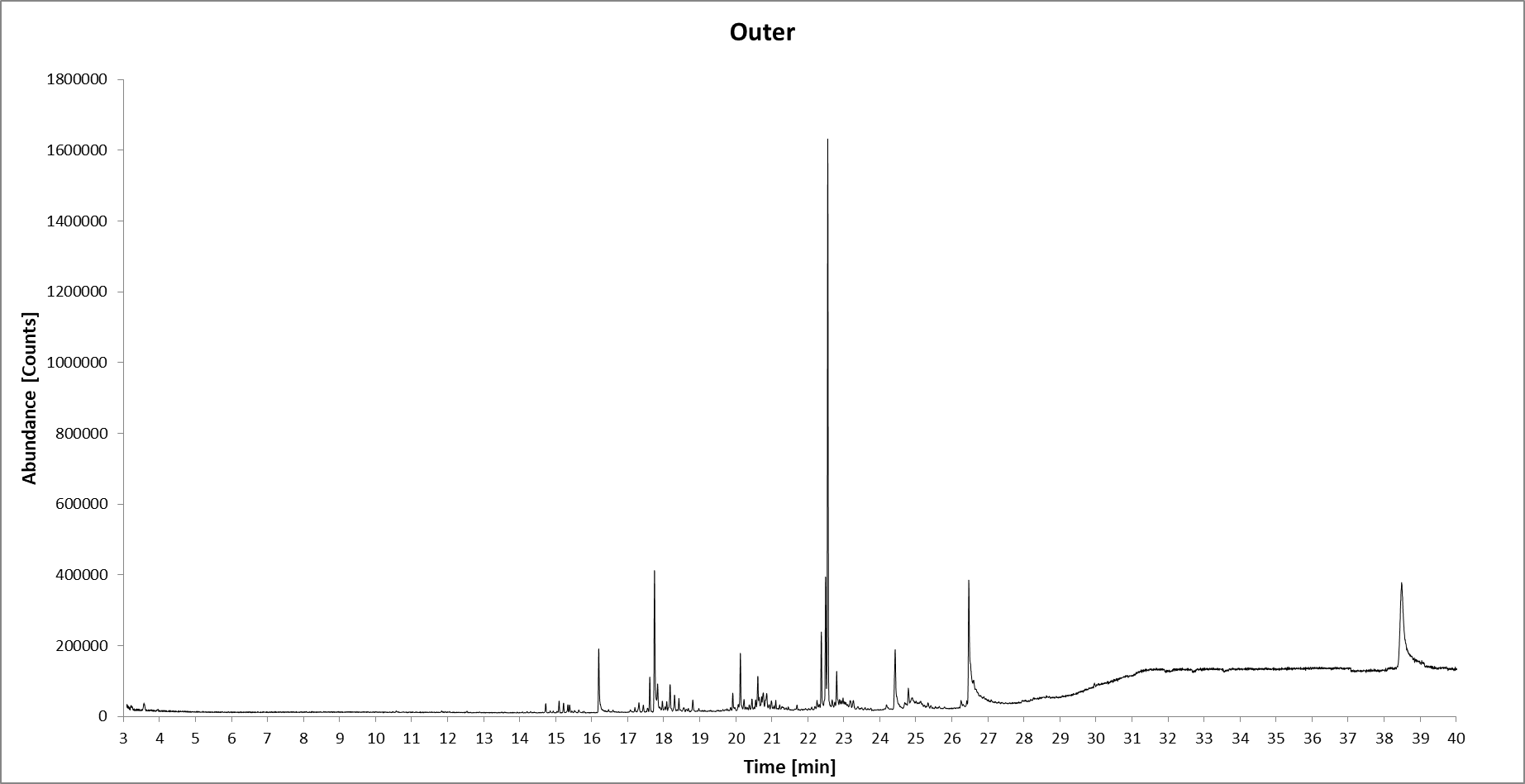


**Figure S6.** GC-MS chromatogram of the extract of the milled outer blue layer of the face mask.

***A review of ecotoxicity data on polypropylene microplastics***

**Table S1.** Search keywords and the number of results obtained. The number of relevant studies is also marked.

|  | **WoS**  (advanced search: articles, category: Environmental Sciences | **ScienceDirect**  (advanced search: Research articles, subject area Environmental Sciences) |
| --- | --- | --- |
| “Microplastics” and “polypropylene” | 688 (20 relevant) | 2003 (14 relevant) |
| “Microplastics” and “polypropylene” and “toxic” | 59 (7 relevant^1^) | 1175 (13 relevant^2^) |

^1^all the same as for the “Microplastics” and “polypropylene” WoS search, ^2^12 of these are the same as for the “Microplastics” and “polypropylene” ScienceDirect search
